# Upregulation of Transient Receptor Potential Canonical Type 3 Channel via AT1R/TGF-*β*1/Smad2/3 Induces Atrial Fibrosis in Aging and Spontaneously Hypertensive Rats

**DOI:** 10.1155/2019/4025496

**Published:** 2019-11-23

**Authors:** Rongfang He, Juan Zhang, Dan Luo, Yiyan Yu, Tangting Chen, Yan Yang, Fengxu Yu, Miaoling Li

**Affiliations:** ^1^Key Laboratory of Medical Electrophysiology of Ministry of Education, Medical Electrophysiology Key Lab of Sichuan Province, Collaborative Innovation Center for Prevention and Treatment of Cardiovascular Disease, Institute of Cardiovascular Research, Southwest Medical University, Luzhou 646000, China; ^2^Infectious Disease Department, The Affiliated Hospital of Southwest Medical University, Luzhou 646000, China; ^3^Affiliated Hospital of Southwest Medical College, Cardiothoracic Surgery, Luzhou 646000, China

## Abstract

Fibroblast proliferation and migration are central in atrial fibrillation (AF) promoting structure remodeling, which is strongly associated with aging and hypertension. Transient receptor potential canonical-3 channel (TRPC3) is a key mediator of cardiac fibrosis and the pathogenesis of AF. Here, we have observed the increased TRPC3 expression that induced atrial fibrosis which possibly is either mediated by the aging process or related to hypertensive progression. In this study, we measured the pathological structure remodeling by H&E staining, Masson staining, and transmission electron microscope (TEM). The protein expression levels of fibrotic biomarkers and TRPC3 were measured by Western blotting with atrial tissues from normotensive Wistar Kyoto rats (WKY 4m-o (4 months old)), old WKY (WKY 24m-o (24 months old)), spontaneously hypertensive rat (SHR 4m-o (4 months old)), and old SHR (SHR 24m-o (24 months old)). To illuminate the molecular mechanism of TRPC3 in atrial fibrosis of aging rats and SHR, we detected the inhibited role of TRPC3 selective blocker ethyl-1-(4-(2,3,3-trichloroacrylamide) phenyl)-5-(trifluoromethyl)-1H-pyrazole-4-carboxylate,pyrazole-3 (Pyr3) on angiotensin II (Ang II) induced fibrosis in neonatal rat atrial fibroblasts. The pathological examination showed that the extracellular matrix (ECM) and collagen fibrils were markedly increased in atrial tissues from aged and hypertensive rats. The protein expressions of fibrotic biomarkers (collagen I, collagen III, and transforming growth factor-*β*1 (TGF-*β*1)) were significantly upregulated in atrial tissues from the WKY 24m-o group, SHR 4m-o group, and SHR 24m-o group compared with the WKY 4m-o group. Meanwhile, the expression level of TRPC3 was significantly upregulated in WKY 24m-o and SHR 4m-o atrial tissues compared to WKY 4m-o rats. In isolated and cultured neonatal rat atrial fibroblasts, Ang II induced the atrial fibroblast migration and proliferation and upregulated the expression levels of TRPC3 and fibrotic biomarkers. TRPC3 selected blocker Pyr3 attenuated the migration and proliferation in neonatal rat atrial fibroblasts. Furthermore, Pyr3 significantly alleviated Ang II-induced upregulation of TRPC3, collagen I, collagen III, and TGF-*β*1 through the molecular mechanism of the TGF-*β*/Smad2/3 signaling pathway. Similarly, knocking down TRPC3 using short hairpin RNA (shTRPC3) also attenuated Ang II-induced upregulation of TGF-*β*1. Pyr3 preconditioning decreased Ang II-induced intracellular Ca^2+^ transient amplitude elevation. Furthermore, AT1 receptor was involved in Ang II-induced TRPC3 upregulation. Hence, upregulation of TRPC3 in aging and hypertension is involved in an atrial fibrosis process. Inhibition of TRPC3 contributes to reverse Ang II-induced fibrosis. TRPC3 may be a potential therapeutic target for preventing fibrosis in aging and hypertension.

## 1. Introduction

Atrial fibrillation (AF) is the most common arrhythmia in clinical practice, and pharmacological approaches have weak effects on AF treatment. One of the crucial reasons is hard to reverse the process of myocardial fibrosis. The structural remodeling is an important contributor to the AF substrates. The structural remodeling caused by atrial fibrosis promotes the occurrence and progression of AF [1, 2]. The incidence of AF increases with age [3, 4]. Hyperactivation of the renin-angiotensin-aldosterone system (RAAS) in hypertension is also closely related to the occurrence of AF [5]. Aging and hypertension both induce atrial fibrosis [6]. Preventing atrial fibrosis may be one of the key targets for the treatment of AF [7].

The hallmark of atrial fibrosis is fibroblast migration, proliferation, and extracellular matrix (ECM) protein deposition. Aging and hypertension are pivotal risk factors for atrial fibrosis. The classic mechanisms of fibroblast proliferation and matrix deposition are involved in the upregulation of collagen I, collagen III, activation of TGF-*β*1, and downstream Smad signaling pathways. Periostin also plays an important role in cardia remodeling [8]. However, the factors that cause the activation of these signaling pathways are varied, such as myocardial ischemia, apoptosis, and activation of the RAAS system. Its mechanism is not fully clarified. Finding targets to inhibit atrial fibrosis is helpful to the treatment and prevention of AF.

Excessive Ca^2+^ influx mediates the process of atrial fibrosis during atrial fibrillation. Canonical transient receptor potential 3 (TRPC3) channel is a non-voltage gated and nonselective cation channel that regulates Ca^2+^ homeostasis. Recently, TRPC3 channel was identified as a mediator of myocardial fibrosis. The electrophysiological data showed [9] that the expression of TRPC3 was increased in fibroblasts from AF patients and a rapid pacing dog model of AF. In a rapid pacing dog model, TRPC3 blockade prevents AF substrate development. However, up to date, it is not clear whether TRPC3 upregulation promotes atrial fibrosis related to aging process or hypertension [10]. Calcium influx through TRPC3 was increased in vascular smooth muscle cells and aortic tissue from SHR compared with WKY. Increased TRPC3 in the vasculature is important for blood pressure regulation. Upregulation of TRPC3 induces in cerebrovascular remodeling during hypertension via transactivation of epidermal growth factor receptor- (EGFR-) dependent signaling pathways [11–13]. Enhancement of TRPC3 activity at the cytoplasmic and mitochondrial levels contributes to redox signaling and calcium dysregulation in the vasculature from SHR [14]. An increase in the molecular coupling between inositol 1,4,5-trisphosphate receptor (IP3R) and TRPC3 augments endothelin-1- (ET-1-) induced vasoconstriction during hypertension [15]. Sierra-Valdez et al. analyzed the structure-function relation of TRPC3 and found that cytoplasmic domain was the target of channel allosteric modulated site [16]. From the above studies, however, it is not illuminated that fibroblasts were activated by TRPC3 during hypertension. Aging also is susceptible to fibrogenesis; the molecular mechanism has not fully illuminated [12]. Here, we undertook this study to test the following hypotheses: (1) the expression level of TRPC3 from atrial fibroblast is upregulated in aging and hypertension, which is involved in atrial fibrosis; (2) TRPC3 was upregulated through TGF-*β*1/Smad signaling pathways to promote atrial remodeling.

## 2. Materials and Methods

### 2.1. Chemicals and Reagents

Ang II (#HY-13948) was purchased from MedChemExpress company (MCE, USA), and Pyr3 (# P0032) and PD123 319 (#P186) were purchased from Sigma-Aldrich (Saint Louis, MO, USA). Collagenase (tape 2) was obtained from Worthington Biochemical. Fetal bovine serum (FBS) was purchased from GIBCO-BRL Life Technologies (Grand Island, NY). Antibodies against TGF-*β*1, GAPDH, and periostin were obtained from Cell Signaling Technology (Beverly, MA, USA). Antibodies against TRPC3 were obtained from Alomone Laboratories (Jerusalem, Israel), and antibodies against collagen I, collagen III, angiotensin II type 1 receptor (AT1R), angiotensin II type 2 receptor (AT2R), Smad2/3, and phosphorylated Smad2/3 were obtained from Abcam (Cambridge, MA, USA). A 24-well transwell system (8.0 *μ*m porous membrane, Corning, NY, USA) was used to measure cell migration.

### 2.2. Experimental Animals

Healthy male normotensive Wistar Kyoto rats (WKY 4m-o, 4 months old), old WKY (WKY 24m-o, 24 months old), spontaneous hypertensive rat (SHR 4m-o, 4 months old), and old SHR (SHR 24m-o, 24 months old) were obtained from the animal care center of Southwest Medical University (Luzhou, China). During the whole experiments, the rats had free access to a standard chow diet and tap water ad libitum and the rats were housed at a constant room temperature (~25°C) and humidity in a 12 h day/12 h night cycles. All animal experiments were performed with the Guide for the Care and Use of Laboratory Animals from the US National Institutes of Health and were approved by the Ethical Committee on Animal Care and Use of Southwest Medical University (Luzhou, China).

### 2.3. Histopathological Examination (H&E Staining and Masson's Staining)

The atrial specimens from WKY and SHR were fixed in 10% PBS neutral formalin, dehydrated, washed, and paraffin-embedded. The paraffin-embedded specimens were sectioned and stained with hematoxylin-eosin staining (H&E staining) and Masson's stain. In H&E-stained atrial tissues, the nucleic acids stain dark blue and the proteins stain red to pink. In Masson-stained atrial tissues, the extracellular matrix collagen was stained blue and cardiomyocytes were stained red.

### 2.4. Transmission Electron Microscopy Examination

WKY and SHR were anesthetized with 1% pentobarbital sodium (40 mg/kg), and the atrial tissues were isolated into 0.5mm^3^ sized small chunks, immediately placed in 4% glutaraldehyde fixed for 2 h (4°C), cleaning the small chunks with PBS (0.1 M) for 3 times, and then fixed with 1% osmium tetroxide for 2 h. The small chunks of atrial tissues were gradient dehydration by ethanol and acetone and epoxy resin immersion, embedding, and polymerization. Preparation of 0.5 *μ*m thickness of semithin slices was used to locate the required atrial myocytes by a light microscope and then made 60 nm thickness of ultrathin slices, stained by uranium acetate and lead citrate. The last step was observed by an electron microscope (type H-7500) and photographed with a Gatan-780 CCD.

### 2.5. Cell Culture

Atrial tissues from neonatal (1~3 days old) Sprague-Dawley rats were excised (15 neonates/preparation) and then washed with phosphate-buffered saline solution (PBS) for 3 times. The atrial tissues were cut into 2~3 chunks and transferred to a 25 ml glass jar with 10 mL Trypsin (EDTA, 0.25%) at 4°C for overnight (12~16 h). After that, added 10 mL Dulbecco's modified Eagle's medium (DMEM) with 10% fetal bovine serum (FBS) into the glass jar, incubated in a 37°C humidified atmosphere containing 5% CO_2_ for 5 min. The supernatant was discarded and subjected to enzymatic digestion for 1 min at 37°C with collagenase II (1 mg/ml) and bovine serum albumin (5 mg/mL, BSA) in PBS. The supernatant was discarded and added with the same enzymatic digested solution (10 mL) for 10 min at 37°C; the supernatant was collected, and the enzyme digestion process was repeated 2 times. All the collected supernatants were transferred to a 50 ml tube for centrifugation for 5 min (1000 rpm). To separate the cardiomyocytes from fibroblasts, the cells were seeded in a six-well culture plate with DMEM and kept in a CO_2_ (5%) incubator for 120 min. The upper layer cardiomyocytes were discarded, and the plate was washed with PBS for 3 times and then kept in an incubator with DMEM (10%FBS) for 24 h. After the fibroblasts were grown to confluence and starved of serum for 12 h, then treatment with two protocols was performed. One of the protocol was separately administered with drug into each well with control (no treatment), Ang II (1 *μ*mol/L), and Ang II with Pyr3 (10 *μ*mol/L) for 48 h, and then the expression levels of protein TRPC3, TGF-*β*1, periostin, collagen I, collagen III, Smad2/3, and p-Smad2/3 were measured. The second protocol was separately administered with control (no treatment), Ang II, Ang II with valsartan (10 *μ*mol/L, Ang II subtype-1 receptor (AT1R) antagonist), and Ang II with PD123 319 (10 *μ*mol/L, Ang II subtype-2 receptor (AT2R) antagonist) for 48 h, and then the expression levels of protein TRPC3 were measured.

### 2.6. Design of TRPC3 RNA Interference (RNAi) Sequence and Construction of Short Hairpin (sh) RNA-Expressing Lentiviral Vectors

Three designed TRPC3-specific sequences were selected to use the online short interfering RNA tools with the reference sequence of TRPC3; the target sequences of TRPC3-1 (5′-GGACAGAAATGCTAATTAT-3′), TRPC3-2 (5′-GAACGAAGGTGAACTGAAA-3′), and TRPC3-3 (5′-GGGTCAAACTTGCCATTAA-3′) were homologous with those of TRPC3-specific mRNA. The sequences of TRPC3-1 are the best ones for TRPC3 RNA interference. Lentiviral vector carrying shRNA TRPC3 gene used the vector aGV248. shRNA TRPC3 was transfected into the cultured fibroblasts according to the manufacturer's instructions. Briefly, when the fibroblasts were confluence to 80% in 6-well plates, respectively added Blank (no Lentiviral vector ) or 15 *μ*l Lentiviral vector (LV-shCtrl), 15 *μ*L carrying shRNA TRPC3 gene Lentiviral vector (LV-shTRPC3, 1E+9TU/mL), and virus transfection strengthening agent (HitransG A & P, 100 *μ*L) to mix together. After 24 hours post transduction, the media mixtures were removed and replaced with DMEM. A fluorescence microscope was used to monitor the transfection efficiency. The fibroblasts were cultured for another 48 hours, and then the protein expression of TRPC3 and TGF-*β*1 was measured.

### 2.7. Cell Proliferation Assays (Wound Healing and Transwell Migration Assays)

In wound healing assay, when the fibroblasts were grown to confluence, a linear wound was made by 1 ml pipette tip cross in the confluent cell layer. The fibroblasts in each well were washed three times to remove detached cells and debris and then treated with Ang II, Ang II with Pyr3, and Pyr3 alone in each well. After that, the wells were imaged at 0 h, 24 h, and 48 h after treatment. The size of wounds was measured by ImageJ software. In transwell assay, after fibroblasts are prepared, the suspension should be diluted to an optimal density for seeding. Transwells are placed on a 24-well plate. The suspension of fibroblasts was added into the transwell with no air bubbles. At the same time, fibroblasts were treated with Ang II, Ang II with Pyr3, and Pyr3 alone in each transwell. Following incubation for 48 h, the membranes of transwells are fixed by dipping in 70% ethanol solution. After drying the membranes, fibroblast staining solution is added for approximately 30 minutes at room temperature. And then, the transwell was washed with PBS. Finally, the membrane can be excised and placed on a microscope slide. Cells on the underside of the membrane represent the number of cells that have migrated.

### 2.8. Ca^2+^ Transient Measurements

Cultured fibroblasts were seeded in 96-well plates. After confluence to 90%, the fibroblasts were used to measure the calcium transients. The fibroblasts were washed twice in PBS and then incubated with intracellular calcium indicator Fura-2-acetoxymethyl (Fura-2/AM, 1 *μ*mol/L, Invitrogen, USA; #F1221) for 30 min at 37°C; at the end of the loading period, the fibroblasts were washed twice with PBS, and intracellular free Ca^2+^ imaging was measured by a cell imaging Multi-Mode reader (Cytation 5, BioTek). Fluorescence of the Fura-2/AM-loaded fibroblasts was measured using two excitation wavelengths: 340 nm and 380 nm, and an emission wavelength: 510 nm. The ratio of 340/380 was calculated, which represents the relative intracellular free Ca^2+^ fluorescence intensity. The experiment protocols were performed with (*n* = 6) or without of Pyr3 (*n* = 6) in Ca^2+^-free Tyrode's solution for 1 min, followed with or without Pyr3 in Ca^2+^-free Tyrode's solution with Ang II (1 *μ*mol/L) for 4 min, and then with or without Pyr3 in Ang II (1 *μ*mol/L) with 1.8 mmol/L Ca^2+^ Tyrode's solution for 4 min.

### 2.9. Quantitative Real-Time PCR (qRT-PCR)

Total RNA was extracted from WKY 4m-o (*n* = 8), WKY 24m-o (*n* = 8), SHR 4m-o (*n* = 8), and SHR 24m-o (*n* = 8) using NucleoZOL reagents (MACHEREY-NAGEL, Germany). The cDNA was generated from 1 *μ*g of total RNA using the ReverTra-Plus kit (TOYOBO), and real-time PCR was carried out using the QuantiNova SYBR Green PCR Kit (QIAGEN, Germany) following the manufacturer's instructions. The relative gene expression levels of the TRPC3, Col1, Col3, and TGF-*β*1 were normalized to the housekeeping gene GAPDH. The mRNA expression levels were calculated by the 2^-*ΔΔ*Ct^ method. The primer sequences used were as follows: Trpc3 forward 5′-GTGTCTGGTCGTGTTGGTCGT-3′, reverse 5′-GATGAAGGAGGCAGCGTGAG-3′; Col1a1 forward 5′-CATGCCGTGACCTCAAGATG-3′, reverse 5′-TGTACCAGTTCTTCTGAGGCACA-3′; Col3a1 forward 5′-AGTGGCCATAATGGGGAACG-3′, reverse 5′-GCTGACCATCTGATCCAGGG-3′; Tgfb1 forward 5′CTGCTGACCCCCACTGATAC-3′, reverse 5′-AGCCCTGTATTCCGTCTCCT-3′; GAPDH forward 5′-CGGCAAGTTCAACGGCACAG-3′, reverse 5′-CGCCAGTAGACTCCACGACAT-3′.

### 2.10. Western Blotting

Proteins were extracted using RIPA buffer (Beyotime) containing protease and phosphatase inhibitors (Roche, Germany). The supernatants were collected after centrifugation at 4°C (12000 rpm, 15 min), and the protein concentration was detected with bicinchoninic acid (BCA assay, Beyotime, China). The protein samples (25 *μ*g) were separated with 10% sodium dodecyl sulfate-polyacrylamide (SDS-PAGE) gel electrophoresis and transferred to polyvinylidene difluoride membranes (PVDF, Bio-Rad Laboratories, Hercules, CA, USA). 5% of nonfat dry milk in TBST was used to block the membrane for 1 h at room temperature. Primary antibodies including TRPC3 (1 : 1000), collagen I (1 : 1000), collagen III (1 : 1000), TGF-*β*1 (1 : 1000), periostin (1 : 1000), AT1R (1 : 1000), AT2R (1 : 1000), GAPDH (1 : 1000), Smad2/3 (1 : 1000), and phosphorylated Smad2/3 (1 : 1000), which were incubated at 4°C overnight. After incubation with primary antibody, the PVDF membranes were washed for 10 min 3 times in TBST. And then the membranes were incubated with horseradish peroxidase-conjugated secondary antibody (1 : 10000, Santa Cruz Biotechnology, Santa Cruz, USA) for 1 hour at room temperature. The immunoblotting bands were shown via an enhanced chemiluminescent detection kit (Gel Imaging System, Bio-Rad). The image was acquired and analyzed by Quantity One Software.

### 2.11. Statistical Analysis

All the data were presented as the mean ± standard deviation (SD). The differences were analyzed by GraphPad prism software 7.0. Quantitative data were analyzed by one-way ANONA tests followed by post hoc analysis. All values with *P* < 0.05 represented a significant difference.

## 3. Results

### 3.1. Animal Characteristics

Detailed animal characteristics are shown in Table 1; there was a significant difference of body weight in WKY 24m-o (*n* = 8, *P* < 0.01) and SHR 24m-o (*n* = 8, *P* < 0.05) compared to WKY 4m-o, and a significant difference of body weight was also shown between WKY 24m-o and SHR 24m-o (*n* = 8, *P* < 0.01). Systolic and diastolic blood pressures were significantly higher in SHR 4m-o and SHR 24m-o compared to WKY 4m-o and WKY 24m-o (*n* = 8, *P* < 0.01). There was no significant difference in heart rate and blood glucose.

### 3.2. Pathological and Ultrastructural Changes in the Atrium from Aging and Hypertensive Rat

**Figure 1:**
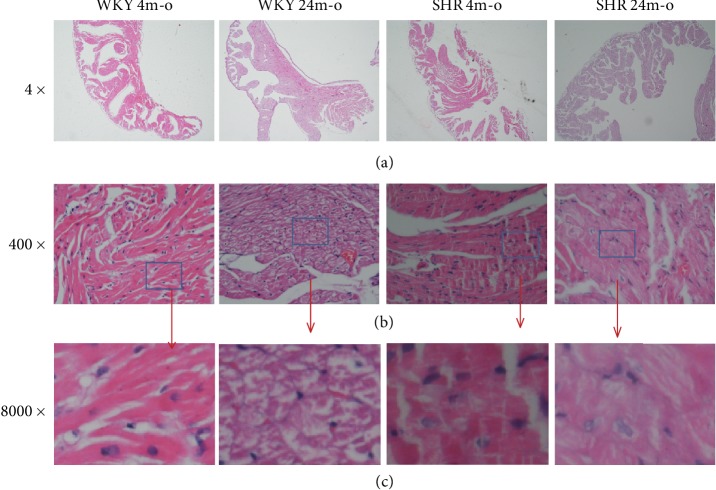
Atrial structural remodeling measured by H&E staining from WKY 4m-o, WKY 24m-o, SHR 4m-o, and SHR 24m-o rats. Representative H&E staining: (a) magnification ×4, (b) magnification ×400, and (c) magnified the blue grid from (b).

**Figure 2:**
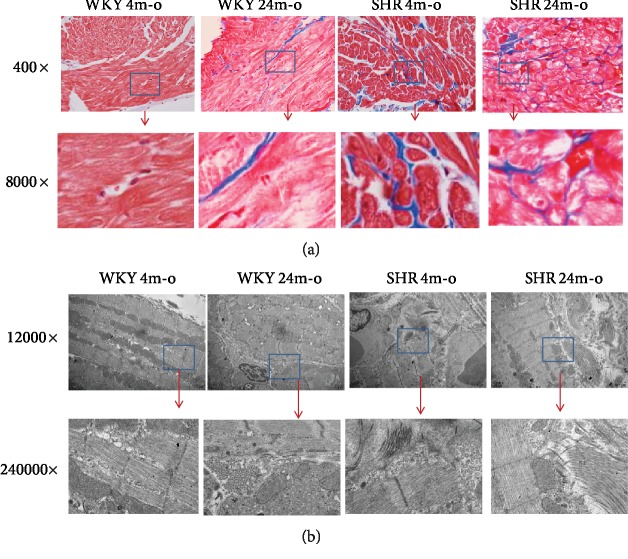
Atrial structural remodeling measured by Masson's staining and TEM in WKY 4m-o, WKY 24m-o, SHR 4m-o, and SHR 24m-o rats. (a) In Masson's staining, the collagen was stained blue, and cardiomyocytes were stained red. In the top panel, magnification is ×400; in the bottom panel, magnification is ×8000, magnifying the blue grid from the top panel. (b) TEM photos: in the top panel, magnification is ×12000; in the bottom panel, magnification is ×240000, magnifying the blue grid from the top panel.

Aging and hypertension are the independent risk factors of atrial structure remodeling. Collagen is the main component of extracellular matrix. In this study, the atrial slices of H&E staining showed significantly increased extracellular matrix (ECM) in atrial tissues from WKY 24m-o, SHR 4m-o, and SHR 24m-o, while rarely ECM could be seen in WKY 4m-o (Figure 1). The cardiac muscles were divided into many branches by ECM. The space between each branch was wider, and the cell nucleus is misaligned. Furthermore, the atrial slice of SHR 24m-o was observed to be significantly misaligned and the muscle was divided into reticular structures (Figure 1(c)). The slices of Masson's staining showed apparent blue color in WKY 24m-o, SHR 4m-o, and SHR 4m-o (shown with arrows, Figure 2(a)). Muscle filament of atrial myocytes was regular organization, but the collagenous fibrils were distinctly observed by TCM in WKY 24m-o, SHR 4m-0, and SHR 4m-0 (Figure 2(b)).

### 3.3. Upregulation of TRPC3 and Fibrotic Biomarkers in the Atrium from WKY and SHR with Aging

**Figure 3:**
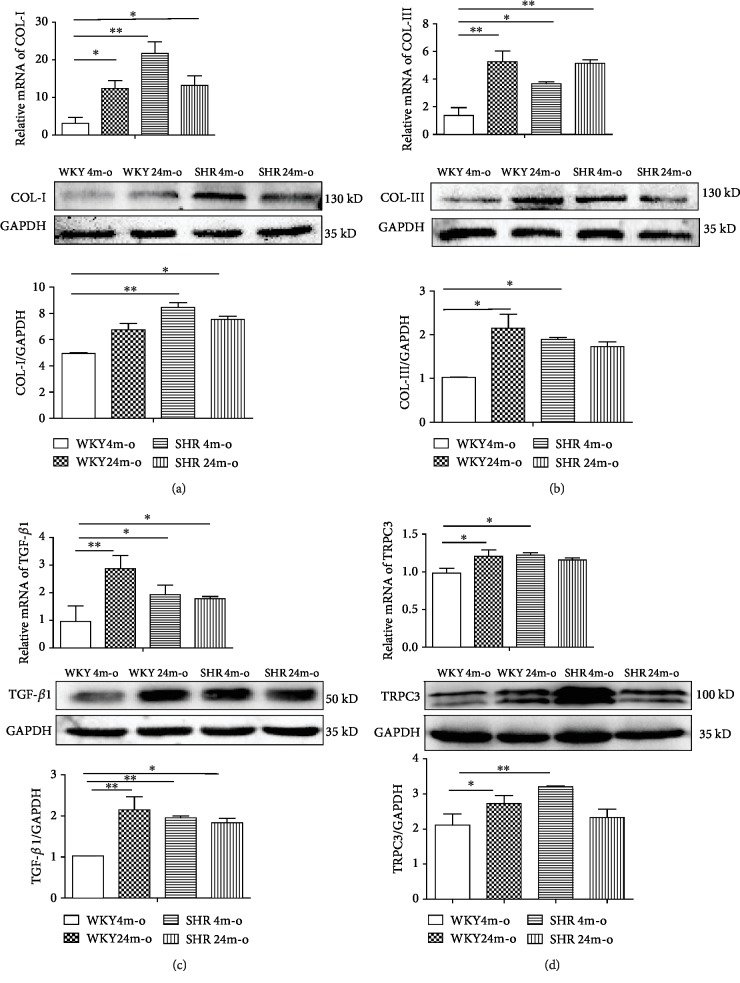
Upregulation of fibrotic biomarkers and TRPC3 in the atrium from SHR and WKY with aging. The relative mRNA and protein expressions of collagen I (left, upper panel, COL-I) (a), collagen III (right, upper panel, COL-III) (b), TGF-*β*1 (left, bottom panel) (c), and TRPC3 (right, bottom panel) (d). All values are presented as the mean ± SD of 8 rats. ^∗^*P* < 0.05 and ^∗∗^*P* < 0.01 significantly different compared with WKY 4m-o.

Morphologically, myocardial fibrosis was found in old WKY, young SHR, and SHR with aging. Fibrotic biomarkers included collagen I, collagen III, and TGF-*β*1; TRPC3 also is associated with fibrosis. The mRNA level of TRPC3 was significantly increased in WKY 24m-o (*P* < 0.05, *n* = 8) and SHR 4m-o (*P* < 0.05, *n* = 8) compared to WKY 4m-o (Figure 3(d)), in accordance with the protein expression level. The mRNA level of COL-1 was significantly increased in WKY 24m-o (*P* < 0.05, *n* = 8), SHR 4m-o (*P* < 0.01, *n* = 8), and SHR 24m-o (*P* < 0.05, *n* = 8) compared to WKY4m-o (Figure 3(a)), and the mRNA level of COL-III was also markedly increased in WKY 24m-o (*P* < 0.01, *n* = 8), SHR 4m-0 (*P* < 0.05, *n* = 8), and SHR 24m-0 (*P* < 0.01, *n* = 8) (Figure 3(b)). The COL-I protein level was significantly increased in all the SHR groups compared to WKY 4m-o (Figure 3(a)). The COL-III protein level was significantly increased in WKY 24m-o and SHR 4m-o (Figure 3(b)). TGF-*β*1 is a fibrosis marker; the mRNA and protein expression levels of TGF-*β*1 were all increased in SHR, WKY, and SHR in aging compared to the WKY 4m-o group (Figure 3(c)). The data indicated that the upregulation of TRPC3 was accompanied with the upregulation of a fibrotic biomarker in aging and hypertension.

### 3.4. TRPC3 Is Involved in Ang II-Induced Cell Migration and Proliferation

**Figure 4:**
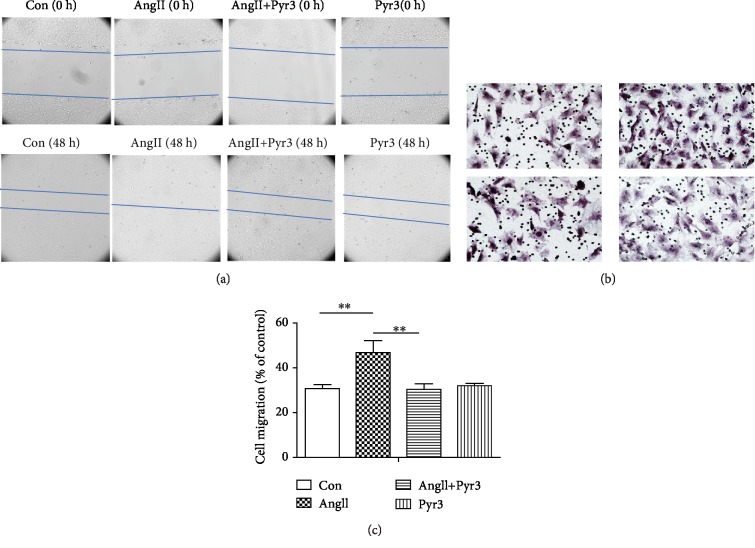
TRPC3 inhibition prevents Ang II-induced cell migration and proliferation. (a). After wound healing assay, the wound of the 24-well cultured confluent neonatal rat atrial fibroblasts was imaged before and after administration of Pyr3 (10 *μ*mol/L) and Ang II with or without Pyr3 (10 *μ*mol/L) for 48 h; the wound edges were imaged under a 10x objective. (b) Accurately measure cell migration by transwell assay. Transwells are placed in wells, and cell suspension is pipetted into the upper chambers, incubating the cells with administration of Pyr3 (10 *μ*mol/L) and Ang II with or without Pyr3 (10 *μ*mol/L) for 48 h; image the migrated fibroblasts from underside membrane, count the migrated cells on a grid under magnification ×200, and count 5 high power fields to calculate the average cell number per field. All data shown are the mean values ± SD and are expressed as % of the control value (first bar). ^∗^*P* < 0.05 versus control (the first ^∗^) and versus Ang II (the second ^∗^).

TRPC3 is upregulation in aging and hypertension. To understand the mechanisms of TRPC3 associated with atrial fibrosis, we treated the neonatal atrial fibroblasts with fibrotic agent Ang II (1 *μ*mol/L) with or without TRPC3 inhibitor Pyr3 (10 *μ*mol/L) for 48 hours to measure the cell migration and proliferation.  Figure 4(a) shows images of the wound at 0 hour and 48 hours. Wound healing assay indicated that Ang II significantly increased the proliferation of fibroblasts compared to the control group and TRPC3 blocker Pyr3 attenuated Ang II-induced proliferation (Figure 4(a), *n* = 5). To accurately calculate the number of migrated fibroblasts, transwell migration assay was used to detect the response. Data calculated the number of fibroblasts migrating outside of the bottom membrane. The result showed that Ang II markedly increased fibroblast migration and Pyr3 decreased the migration under the condition of Ang II stimulation (*n* = 4, *P* < 0.01). The two methods demonstrated that Pyr3 prevents Ang II-induced fibrotic response in fibroblasts.

### 3.5. Role of TRPC3 Selected Blocker Pyr3 in the Intracellular Ca^2+^ Response Induced by Ang II

**Figure 5:**
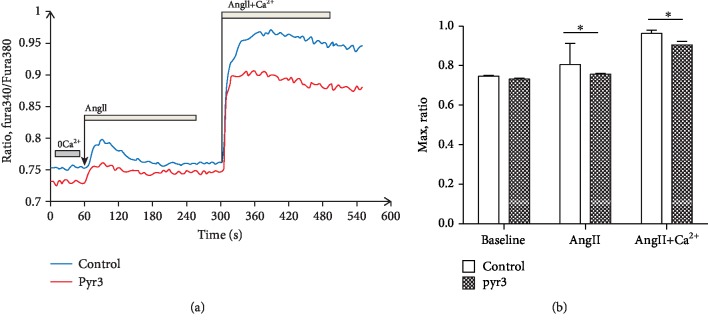
Intracellular Ca^2+^ rises induced by Ang II with or without extracellular Ca^2+^. Intracellular free Ca^2+^ detected by ratio values of Fura340/380. During the fluorescence calcium acquisition process, in the control group, fibroblasts were perfused with Ca^2+^-free Tyrode's solution for 1 minute, following perfusion with Ca^2+^-free Tyrode's solution with Ang II (1 *μ*mol/L) for 4 minutes, and then perfused with 1.8 mmol/L Ca^2+^ Tyrode's solution with Ang II (1 *μ*mol/L) for 4 minutes. In the Pyr3 group, fibroblasts were perfused with Ca^2+^-free Tyrode's solution with Pyr3 (10 *μ*mol/L) for 1 minute, following perfusion with Ca^2+^-free Tyrode's solution with Ang II (1 *μ*mol/L) for 4 minutes containing Pyr3 (10 *μ*mol/L), and then perfused with 1.8 mmol/L Ca^2+^ Tyrode's solution with Ang II (1 *μ*mol/L) for 4 minutes containing Pyr3 (10 *μ*mol/L). (a) The changes of fluorescence calcium microphotographs, traces, and quantitative ratio values. (b) The statistical graph of fluorescence calcium ratio values. Baseline bar shows the mean values of Ca^2+^-free Tyrode's solution, Ang II bar shows the peak values of calcium transient of Ca^2+^-free Tyrode's solution with Ang II (1 *μ*mol/L), and Ang II+Ca^2+^ bar shows the peak values of calcium transient with 1.8 mmol/L Ca^2+^ Tyrode's solution with Ang II (1 *μ*mol/L).

When cultured cardiac fibroblasts were treated in a calcium-free medium (Figures 5(a) and 5(b)), the resting calcium ratio values (ratio, Fura340/Fura380) in control (0.746 ± 0.001, *n* = 6) were higher than those in Pyr3 preconditioning (0.732 ± 0.006), but data showed no significantly statistical differences. Application of Ang II (1 *μ*mol/L) in the calcium-free solution evoked a calcium transient in both control and Pyr3 preconditioning. The data showed that Pyr3 significantly decreased calcium transient under the condition of calcium-free solution (*P* < 0.05, *n* = 6), and then solution was changed to 1.8 mmol/L CaCl_2_ with 1 *μ*mol/L Ang II; the calcium transient was significantly more enhanced in both control and Pyr3 preconditioning, while the calcium transient in Pyr3 preconditioning is significantly lower than that in control (*P* < 0.05, *n* = 6).

### 3.6. Suppression of TRPC3 Prevents Ang II-Induced Deposition of Extracellular Matrix Proteins

**Figure 6:**
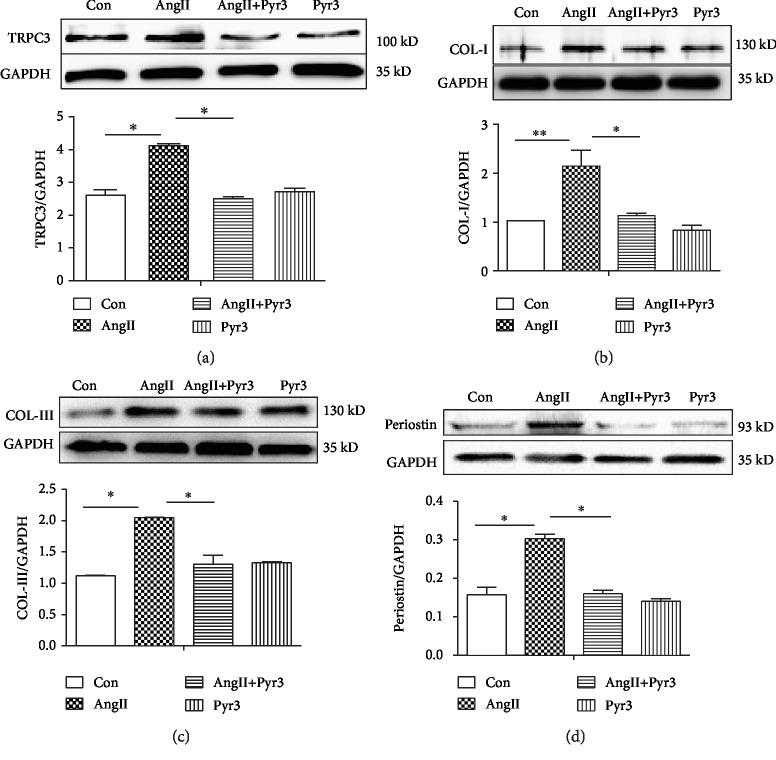
TRPC3 inhibition prevents Ang II-induced deposition of extracellular matrix protein. In cultured fibroblast, administrated Ang II (1 *μ*mol/L) with or without Pyr3 (10 *μ*mol/L) to measure the protein expression of TRPC3 (a), COL-1 (b), COL-III (c), and periostin (d) by Western blotting. The ratio of TRPC3, COL-I, COL-III, and periostin normalized to GAPDH was calculated. The analyzed data was shown from 5 independent experiments. All data presents the mean values ± SD. ^∗^*P* < 0.05 shows a significant difference from control or from treatment with Ang II.

The above data showed that the expression of TRPC3 was elevated in the atrium from aging and hypertensive rats. Furthermore, the inhibition of TRPC3 significantly attenuated fibroblast migration and proliferation, but the exact mechanism is unclear. So, the contribution of TRPC3 on atrial fibrosis was further explored in cultured fibroblasts from neonatal rat atrial tissues, after the fibroblasts were grown to confluency to 70-80% of 6-well plate. In the treatment of Ang II (1 *μ*mol/L) with or without Pyr3, or Pyr3 alone for 48hours, the experiment was repeated five times. 48 h later, the cells were obtained to detect the protein level by Western blotting. In Figure 6, compared with the control group, the protein level of TRPC3 was significantly increased under the condition of Ang II stimulation, while Pyr3 inhibited Ang II-induced upregulation of TRPC3. In the treatment of the fibroblasts with Pyr3 alone, the expression of TRPC3 was unchanged. The data suggested that Ang II stimulation could upregulate the expression of TRPC3. On the normal condition, TRPC3 shows low expression level in fibroblasts (Figure 6(a), *n* = 6). Furthermore, Ang II significantly upregulated the expression levels of fibrotic biomarkers COL-1 and COL-III (Figure 6(b), *n* = 6, *P* < 0.01; Figure 6(c), *n* = 6, *P* < 0.05). Moreover, Pyr3 reversed Ang II-induced upregulation of COL-I (Figure 6(b), *n* = 6, *P* < 0.05) and COL-III (Figure 6(c), *n* = 6, *P* < 0.01). Periostin is produced by myocardial fibroblasts and also a mediator in cell-matrix crosstalk association with fibroproliferative diseases in the myocardium. In cultured fibroblasts, the expression of periostin was elevated by Ang II. Pyr3 prevents Ang II-induced upregulation of periostin. Taken together, the data showed that the inhibition of TRPC3 attenuated the deposition of extracellular matrix protein.

### 3.7. Downregulation of TRPC3 by LV-shTRPC3 Suppresses the Protein Expression of TGF-*β*1

**Figure 7:**
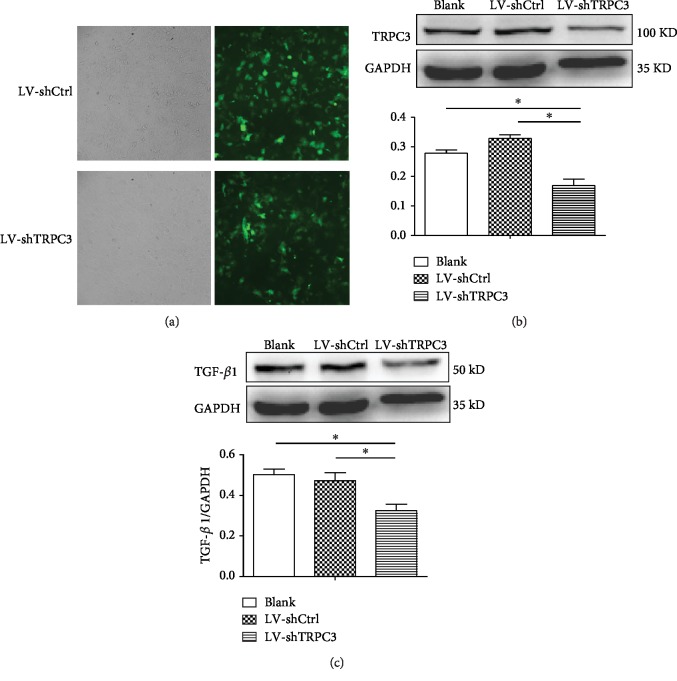
Downregulation of TRPC3 by LV-shTRPC3 suppressed the protein expression of TGF-*β*1. (a) GFP expression in fibroblasts under a fluorescence microscope after 72 h of infection. (b) The protein expression by Western blot analysis of TRPC3 in Blank, LV-shCtrl, and LV-shTRPC3. (c) The protein expression by Western blot analysis of TGF-*β*1 in Blank, LV-shCtrl, and LV-shTRPC3 (*n* = 5). ^∗^*P* < 0.05 shows a significant difference from Blank or LV-shCtrl with LV-shTRPC3.

TGF-*β*1 is a pivotal biomarker of fibrosis. To further define the role of TRPC3 involved in myocardial fibrosis, lentiviral vector carrying shRNA TRPC3 gene was used to simulate the similar effect of TRPC3 selected blocker. In Figure 7, Blank indicated that no lentiviral vector was added in fibroblasts, shCtrl indicated that lentiviral vector treated the fibroblasts with no shTRPC3, and LV-shTRPC3 indicated that the fibroblasts transfected with lentiviral vector carrying shRNA TRPC3. Data showed that Blank and shCtrl had no effect on the expression of TRPC3 and TGF-*β*1; however, shTRPC3 induced significantly the downregulation of the expressions of both TRPC3 (Figure 7(b)) and TGF-*β*1 (Figure 7(c)). It means that the downregulation of TRPC3 is involved in decreasing TGF-*β*1 expression.

### 3.8. Ang II-Induced TRPC3 Upregulation through AT1 Receptor

**Figure 8:**
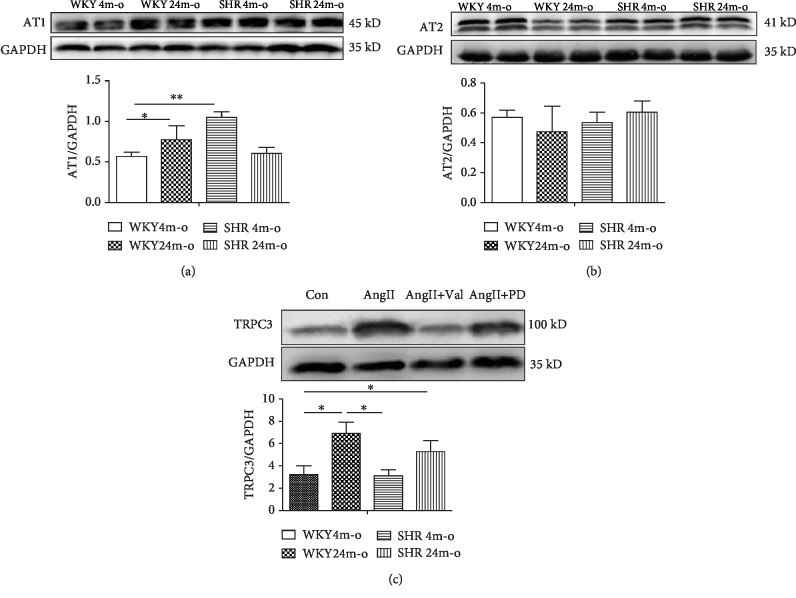
AT1R blocker inhibited Ang II-induced TRPC3 upregulation. The protein expressions of ATR1 (a) and AT2R (b) are measured by Western blotting from WKY and SHR with or without aging. (c) The protein expression of TRPC3 was measured by after treatment with Ang II (1 *μ*mol/L), Ang II with AT1R blocker valsartan (Val, 10 *μ*mol/L), or Ang II with AT2R blocker PD123 319 (PD, 10 *μ*mol/L) for 48hours. The analyzed data was shown from 5 independent experiments. All data presents the mean values ± SD. ^∗^*P* < 0.05 shows a significant difference from control or from treatment with Ang II.

Ang II plays a key role in the modulation of many functions through strong affinity to the angiotensin II type 1 receptor (AT1R) and angiotensin II type 2 receptor (AT2R). Whether the role of Ang II acts on TRPC3 through its receptor remains unclear. First, we detected the expression levels of AT1R and AT2R in WKY and SHR with or without aging. Compared to the WKY 4m-o group, the expression level of AT1R was apparently increased in WKY 24m-o (Figure 8(a), *n* = 8, *P* < 0.05) and SHR 4m-o (Figure 8(a), *n* = 8, *P* < 0.01). But there was no significant difference between the WKY 4m-o and SHR 24m-o groups (Figure 8(a), *n* = 8, *P* > 0.05). No significant difference was found in AT2R expression from the four groups. We speculate that AT1R may be the key protein to regulate the function of TRPC3. In order to determine the effects, we measured the role of AT1R blocker valsartan (10 *μ*mol/L) and AT2R blocker PD123 319 (10 *μ*mol/L) on TRPC3 expression. In cultured neonatal rat atrial fibroblasts, Western blotting data showed that valsartan inhibited the upregulation of TRPC3 induced by Ang II, while PD123 319 has no action on the upregulation induced by Ang II. The results in Figure 8 demonstrated that Ang II may regulate the function of TRPC3 by AT1R, while not by AT2R.

### 3.9. TRPC3 Blockade Suppresses the TGF-*β*1/Smad2/3 Signaling Pathway

**Figure 9:**
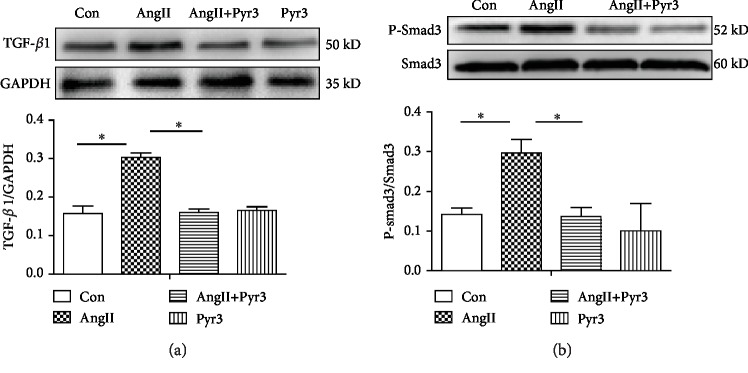
Suppression of the TGF-*β*1/Smad2/3 signaling pathway with TRPC3 blockade. The atrial fibroblasts were exposed to Ang II with or without Pyr3. The protein expressions of TGF-*β*1 (a), P-Smad2/3, and Smad2/3 (b) are measured by Western blotting. The ratio of TGF-*β*1, Smad2/3, and P-Smad2/3 normalized to GAPDH was calculated. The ratio of P-Smad2/3/Smad2/3 shows the phosphorylation state. The analyzed data was shown from 5 independent experiments. All data presents the mean values ± SD. ^∗^*P* < 0.05 shows a significant difference from control or from treatment with Ang II.

TGF-*β*1 plays a key role in atrial fibrosis. Therefore, TGF-*β*1 may be involved in Ang II-induced upregulation of TRPC3. In cultured neonatal rat atrial fibroblasts, with treatment of Ang II with or without Pyr3, the expression of TGF-*β*1 was significantly increased with Ang II stimulation. As we speculated, Pyr3 attenuated the upregulation induced by Ang II treatment. Pyr3 alone had no effect on the expression of TGF-*β*1 (Figure 9(a)). TGF-*β*1/Smad3 is the critical signaling pathway in fibrosis. Therefore, the expression of Smad2/3 and phosphorylation of Smad2/3 (p-Smad2/3) were determined. The results showed that the total protein of Smad2/3 was no apparent different treatment by Ang II with or without Pyr3. However, the P-Smad2/3 and the ratio of P-Smad2/3/Smad2/3 were significantly attenuated induced by Pyr3. As a whole, these results demonstrated that Pyr3 inhibited the atrial fibrosis through TGF-*β*1/Smad2/3 signaling pathway.

## 4. Discussion

Atrial fibrosis induces structural remodeling which is a substrate of AF occurrence and persistence. Aging and hypertension are known risk factors for cardiovascular disease, especially during AF [17]. TRPC3 is a nonselective Ca^2+^ permeant cation channel, which mediates a calcium influx involved in cardiac fibrosis [18]. Some studies showed that TRPC3 was increased in vasculature [10] and peripheral blood monocytes in hypertension [19]. To date, no study has investigated the fibrotic response of atrial fibroblast TRPC3 underlying aging and hypertension. The aim of this study is to elucidate the mechanism of TRPC3 upregulation-induced fibrosis in aging and hypertension. The present study showed that fibrosis was evidenced in the atrium during aging and hypertension. Pathological changes measured by a light microscope and a transmission electron microscope showed an excessive deposition of extracellular matrix. In addition, atrial TRPC3 expression was upregulated in the atrium from SHR, aged WKY and SHR. To mimic this pathological state in cultured atrial fibroblasts, Ang II stimulation plays this effect. Ang II treated the fibroblasts with or without TRPC3 channel selected blocker Pyr3. The results showed that Pyr3 could attenuate the migration, proliferation, and deposition of profibrotic biomarker proteins induced by Ang II. Furthermore, Pyr3 prevented Ang II-induced atrial fibrosis via AT1R involved in the downregulation of the TGF-*β*1/Smad2/3 signaling pathway.

TRPC3 has been implicated in many cardiovascular diseases, such as myocardial hypertrophy [20], heart failure [18, 20], myocardial infarction [21], and AF [22]. The known main mechanism is involved in the calcineurin/NFAT signaling pathway to promote Ca^2+^ influx via TRPC3 and induces mechanical stress, hypertrophy, and heart failure. These studies focused on the profibrotic effects on cardiomyocytes or the cells from other organs or systems; there are seldom papers that reported the effects on fibroblasts. Actually, even more importantly, the pathophysiology action of TRPC3 is involved in cardiac fibroblasts directly inducing migration and proliferation. AF increases with age and induces age-dependent atrial fibrosis. Harada et al. reported that TRPC3 regulated cardiac fibroblast proliferation by controlling Ca^2+^ influx via the extracellular signal regulated kinase (ERK) signaling pathway to upregulate the expression of TRPC3 in vivo AF dog model [9]. We also demonstrated that Pyr3 significantly decreased the calcium transient amplitude in Ang II with extracellular Ca^2+^-free or 1.8 mmol/L Ca^2+^. Aging and hypertension are the key factors to promote atrial fibrosis and eventually lead to AF. Protein aggregation is a hallmark for aging. Ayyadevara et al. [23] first reported that protein aggregation existed during natural aging, chronic hypertension, and myofibroblast aging in vitro [24]. Ang II-induced hypertension shows many of the same aggregated characteristics seen in natural aging, such as fibroblast proliferation and migration, and leads to fibrosis. Hypertensive and aged hearts show similar dysfunction of the heart and related most likely to the development of fibrosis. Based on these proteomic studies, hypertension appears to accelerate or mimic some aspects of natural physiologic aging. Furthermore, young individuals with hypertension exhibit arterial changes similar to those in older persons with normal blood pressure [25, 26]. The same from the abovementioned, in this study, we observed the similar fibrosis features by a light microscope (H&E staining, Masson's staining) and a transmission electron microscope. The atrial tissues from WKY 24m-o, SHR 4m-o, and SHR 24m-o rats all showed significant deposition of ECM and proliferation. In observing the ultrastructure of atrial myocytes under transmission electron microscopy, there were obvious collagenous fibrils between cells. And then, we also measured significant upregulation of protein expression, such as TRPCR3, profibrotic COL-I, COL-III, periostin, and TGF-*β*1 in young SHR, aged WKY, and aged SHR compared to young WKY [27]. It means that aging, the same as hypertension, induced upregulation of TRPC3 accompanied with excessive deposition of extracellular matrix. Our studies demonstrated that atrial TRPC3 expression was upregulated during aging and hypertension, which was accompanied with the enhancement of fibrotic markers.

However, in Figures 4(b) and 4(d), data showed that compared with the WKY 4m-o group, the protein expressions of COL-I and TRPC3 in the SHR 24m-o group were not significantly increased compared with those in the SHR 4m-o group and WKY 24m-o group. The possible reason is that it is not sensitive to response to higher blood pressure, especially response to Ang II in aged hypertensive rats. Myocardial fibrosis already exists in elderly hypertensive rats, so they are less sensitive to changes in blood pressure.

Researchers also found that upregulation of TPRC3 enhances EGFR transactivation, which is involved in hypertension-induced cerebrovascular remodeling by the signaling pathway TRPC3/ADAM17 [11, 13, 28]. Increased proportion and assembly of TRPC3 and TRPC6 contribute to cell depolarization in hypertensive mesenteric vascular smooth muscle cells [29]. In SHR, elevation coupling between type 1 inositol 1,4,5-trisphosphate receptor (IP3R) and TRPC3 enhances cell contraction of mesenteric arteries [15]. TRPC3 also is involved in regulating mitochondrial function and reactive oxygen species (ROS) generation [30, 31]. In SHR, enhancement of TRPC3 activity at the cytoplasmic and mitochondrial levels contributes to Ca^2+^ dysregulation and redox signaling activity in the vasculature [14]. Additionally, excessive migration of monocytes in essential hypertension is related to upregulation of TRPC3 [19, 32]. These research data demonstrated that TPRC3 is increased in mesenteric arteries, cerebrovasculature, mitochondria, and monocytes, which is consistent with our results in atrial tissues from young SHR, WKY, and SHR in aging.

Excessive activation of fibroblasts is the most direct cause of myocardial fibrosis. Fibrosis also is a feature of AF. Hypertension is susceptible to AF occurrence. TRPC3 blockade prevents AF substrate development in an AF model. TRPC3 of fibroblasts plays a pivotal role in AF process via the ERK/microRNA-26/NFAT (nuclear factor of activated T-lymphocytes) signaling pathway. The data indicated that TRPC3 may be a novel potential therapeutic target for AF-induced fibrosis [9]. Additionally, Saliba et al. reported that TRPC3 presented in renal fibroblasts and control fibroblast proliferation and activation through the Ca^2+^-mediated ERK signaling pathway [33]. Our data also implied that the TRPC3 was increased in the atrium from aging and hypertensive rats and accompanied with profibrotic protein upregulation.

RAAS activity plays an important role in functional and structural changes of vasculature that occurs with aging and hypertension [34, 35]. RAAS is involved in the aging and hypertension process. Angiotensin-converting enzyme inhibitor (ACEI) and AT1 receptor blocker could decrease age-associated vascular damage. To understand the mechanism of the relation between upregulated TRPC3 and atrial fibroblasts under aging and hypertension, the cultured neonatal atrial fibroblasts were treated with Ang II with or without TRPC3 inhibitor Pyr3. The data showed that Ang II increased the proliferation, migration, and expression of TRPC3, COL-I, COL-III. Pyr3 prevents Ang II-induced TRPC3 upregulation and profibrotic collagen deposition. It confirmed that Ang II mimics the response of aging and hypertension. TRPC3 also is involved in the proliferation and migration in atrial fibroblasts.

In fact, it is not clear how Ang II regulates the activity of TRPC3. In general, Ang II possesses a physiological function through angiotensin-converting enzymes or by acting through receptors. TRPC3 is a receptor-operated channel modulated by Gq PCRs; the activation of AT1R may induce cation influx via TRPC3 or TRPC6 in mouse cardiomyocytes [32]. The results demonstrate that Ang II promotes the slow increase in the Ca^2+^ transient; TRPC3 and PLC inhibition significantly suppressed the increase of intracellular Ca^2+^ induced by both the sustained stretch and Ang II. The data indicate that the activation of TRPC3/6 by sustained stretch is mediated via the signaling pathway AT1R-Gq-PLC-DAG [36–38]. In cultured fibroblasts, in this study, Ang II treatment with AT1R inhibitor valsartan or AT2R inhibitor PD123 319 was used. Valsartan significantly inhibits Ang II-induced TRPC3 enhancement, while PD123 319 has no remarkable action on TRPC3 expression. Our experiment demonstrated that Ang II upregulated TRPC3 via AT1R, which is consistent with the above study by Yamaguchi et al. in isolated mouse ventricular myocytes.

**Figure 10:**
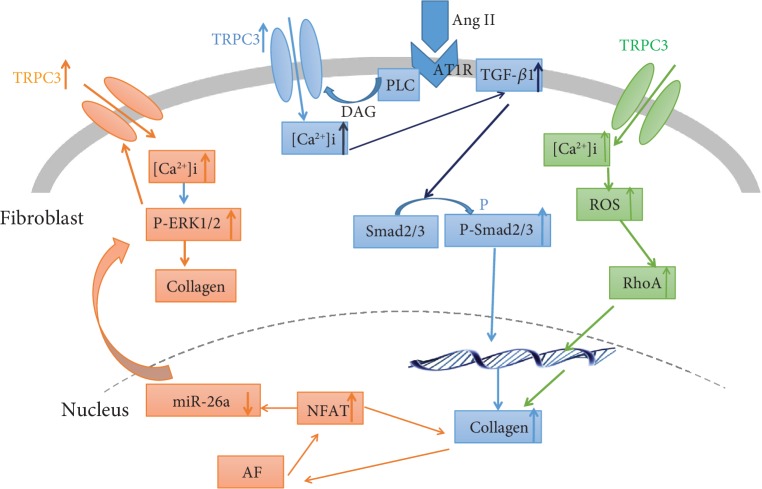
The summary of the signaling pathway of TRPC3 upregulation in fibroblasts involved in fibrosis. The blue signaling pathway is evidenced by this study, and the red and green ones were reported by published papers.

TGF-*β*1 is a central mediator of fibrosis. Ang II is involved in fibrosis via the TGF-*β*1/Smad3 signaling pathway. Cytoskeleton, such as microtubule, is associated with Rho guanine nucleotide exchange factor (GEF-H1), which is involved in TRPC3-mediated RhoA activation induced by mechanical stress in cardiomyocytes and transforming growth factor (TGF-*β*) stimulation in cardiac fibroblasts [39]. TRPC6 interaction with TRPC3-NOX2 protein complex attenuates the hyperglycemia-induced heart failure in mice [18]. TRPC3 inhibition or Ca^2+^ removal inhibits renal fibroblast proliferation and myofibroblast differentiation by suppressing the phosphorylation of extracellular signal regulated kinase (ERK1/2) in cultured fibroblasts. In our study, Pyr3 inhibited Ang II-induced TGF-*β*1 upregulation. Furthermore, lentiviral vector carrying shRNA TRPC3 gene also inhibited TGF-*β*1 expression. In addition, Pyr3 also inhibited the expression of phosphorylated Smad2/3. Our results demonstrated that Pyr3 prevented Ang II-induced atrial fibrosis by the TGF-*β*1/smad2/3 signaling pathway.  Figure 10 summarizes the signaling pathway of the TRPC3 in fibroblasts, which are involved in myocardial fibrosis. The blue one is the signaling pathway evidenced in this study, and the red and green ones were reported by published papers.

## 5. Conclusions

TRPC3 is increased in the atrium from aging and hypertensive rats accompanied with atrial fibrosis. The upregulation of TRPC3 from atrial fibroblasts is involved in fibrosis via the AT1R/TGF-*β*1/p-Smad2/3 signaling pathway. TRPC3 may be a potential therapeutic target under the pathophysiology states such as aging and hypertension.

## Figures and Tables

**Table 1:** Body weight, blood pressure, heart rate and blood glucose from WKY and SHR with or without aging.

	WKY 4m-o (*n* = 8)	WKY 24m-o (*n* = 8)	SHR 4m-o (*n* = 8)	SHR 24m-o (*n* = 8)
Weight	325.00 ± 34.33	407.50±41.74^∗∗^	328.75 ± 38.79	358.12±16.46^∗,##^
Systolic blood pressure (mmHg)	113.87 ± 13.59	134.00 ± 7.34	189.62±23.79^∗∗,##^	184.87±19.72^∗∗,##^
Diastolic blood pressure (mmHg)	91.75 ± 26.47	94.25 ± 10.87	141.12±16.64^∗∗,##^	130.37±18.39^∗∗,##^
Heart rate (bmp/min)	356.12 ± 56.12	345.17 ± 36.17	389.28 ± 32.16	357.54 ± 43.17
Blood glucose (mmol/L)	6.05 ± 0.86	6.75 ± 1.29	6.21 ± 1.53	6.85 ± 1.30

Each value represents the mean ± SD; *n* = 8 rats per group. ^∗^*P* < 0.05 and ^∗∗^*P* < 0.01 compared to WKY 4m-o; ^#^*P* < 0.05 and ^##^*P* < 0.01 compared to WKY 24m-o.

## Data Availability

The data used to support the findings of this study are available from the corresponding authors upon request.
